# “Data safety and monitoring board in non-industry trials: learning it the hard way”

**DOI:** 10.1186/s12931-015-0222-6

**Published:** 2015-05-28

**Authors:** A. Hazenberg, H. A. M. Kerstjens, P. J. Wijkstra

**Affiliations:** Department of Pulmonary Medicine and Tuberculosis, University of Groningen, University Medical Center Groningen, AA11, Hanzeplein 1, 9700 RB Groningen, The Netherlands; Department of Home Mechanical Ventilation, University of Groningen, University Medical Center Groningen, AA62, Hanzeplein 1, 9700 RB Groningen, The Netherlands; Groningen Research Institute for Asthma and COPD, University of Groningen, University Medical Center Groningen, Hanzeplein 1, 9700 RB Groningen, The Netherlands

In the majority of studies, no Data and Safety Monitoring Board (DSMB) is either needed or instituted. We report an investigator initiated study where we should have done this earlier than we did and discuss the lessons we learned.

The EOLUS study was a single center, randomized controlled trial of the initiation of chronic home mechanical ventilation (HMV) at home. Typical indications for HMV are neuromuscular diseases such as amyotrophic lateral sclerosis (ALS) and Duchenne's disease next to thoracic cage deformities. The study was set up to investigate whether the initiation of HMV at home with the help of telemonitoring was not inferior to our usual in hospital start. The primary outcome measure was change in arterial carbon dioxide pressure from baseline to 6 months, for which we calculated a necessary sample size of 52 evaluable patients. The study was performed by an experienced nurse practitioner, as part of a PhD project. Weekly informal supervision was done by a senior pulmonologist and monthly progress meetings were held in a larger group. At these meetings, inclusion, lists of those excluded, and dropouts with their reasons and all deaths were discussed and minutes were always made and distributed. At four occasions over the first 14 months, a death of a patient, all with ALS, was discussed. The number of deaths occurring in this severely ill patients group, nor the circumstances were deemed remarkable. After the inclusion of 33 patients, all events were totaled and presented as such. We were shocked to realize that all 4 patients had died in the home group and none in the hospital group. The study was immediately put on hold and we reported this to the medical ethics committee. The ethics committee requested detailed reports of all cases, and an independent view from experts not involved in the study, including a statistical analysis. The conclusion of the expert group was that the four patients had died due to the progression of their ALS, without an identifiable link to the intervention. They reported no lack of effectiveness in the survivors nor safety problems. The total number of deaths was deemed within the expected rates, but non-normally distributed. The ethics committee accepted the interpretation of the expert group, and decided to restart the study, with the requirement to institute a DSMB. All future severe adverse events were to be presented to the ethics committee immediately, as were the monthly progress reports of the DSMB.

The study was subsequently finished as planned, with 77 patients randomized of which 30 with ALS. At the end, 5 patients in the home care and 2 patients in the hospital group had died, all with ALS. The hypothesis of non-inferiority of HMV institution at home with the help of telemonitoring was endorsed [1].

What did we learn? Most of all we came to fully realize that doing any study in patient groups with a high a priori risk of serious adverse events and especially of deaths, a DSMB with clear pre-set guidelines, meeting frequencies, minutes, and reporting lines to the ethics committee should be in place. In our group, in investigator initiated studies, we had no rulings in place to see to the institution of a DSMB. We now firmly believe such a DSMB should be operational in all studies with high risk interventions. That is already frequently the case in long running pharmacy sponsored studies, and slightly less frequently in device company sponsored studies; investigator initiated studies are probably the biggest omission.

The second lesson we learned is that we should have been keen on seeing at each meeting summary tables of all events having occurred. We did discuss all deaths, but became aware of the bigger, aggregate picture later than we should have.

The DSMB consisted of a clinician expert in the field of chronic ventilation, a statistician and a clinical expert with expertise in clinical trials and methodology, all without any involvement in the conduct of the study. The DSMB evaluated the progress of the trial every four months and if necessary earlier depending on the monthly reports.

In conclusion we believe that it is of great importance that a DSMB is involved in clinical trials not only with high risk interventions but also with high a priori risk of death due to disease under study. If the DSMB had been installed from the start of our study there would have been no reason to put the study on hold. Nevertheless, we learned greatly from the events and nowadays it is mandatory in our department that clinical trials with high risk of death by the intervention or the disease, must have an actively functioning DSMB.
